# DCUN1D1 Is an Essential Regulator of Prostate Cancer Proliferation and Tumour Growth That Acts through Neddylation of Cullin 1, 3, 4A and 5 and Deregulation of Wnt/Catenin Pathway

**DOI:** 10.3390/cells12151973

**Published:** 2023-07-31

**Authors:** Akhona Vava, Juliano D. Paccez, Yihong Wang, Xuesong Gu, Manoj K. Bhasin, Michael Myers, Nelson C. Soares, Towia A. Libermann, Luiz F. Zerbini

**Affiliations:** 1 Cancer Genomics Group, International Centre for Genetic Engineering and Biotechnology, Cape Town 7925, South Africa; akhonavava@gmail.com (A.V.); juliano.paccez@icgeb.org (J.D.P.); 2 Division of Chemical and Systems Biology, Department of Integrative Biomedical Sciences, University of Cape Town, Cape Town 7925, South Africa; 3 Department of Pathology and Laboratory Medicine, Warren Alpert School of Medicine, Brown University, Providence, RI 02912, USA; yihong_wang@brown.edu; 4 BIDMC Genomics, Proteomics, Bioinformatics and Systems Biology Center, Beth Israel Deaconess Medical Center and Harvard Medical School, Boston, MA 02215, USA; xgu@bidmc.harvard.edu (X.G.); tliberma@bidmc.harvard.edu (T.A.L.); 5 Department of Pediatrics Bioinformatics, Emory University School of Medicine, Atlanta, GA 30322, USA; manoj.bhasin@emory.edu; 6 Protein Networks Group, International Centre for Genetic Engineering and Biotechnology (ICGEB), 34149 Trieste, Italy; myers@icgeb.org; 7 Department of Medicinal Chemistry, College of Pharmacy, University of Sharjah, Sharjah P.O. Box 26666, United Arab Emirates; nsoares@sharjah.ac.ae; 8 Research Institute of Medical and Health Sciences, University of Sharjah, Sharjah P.O. Box 26666, United Arab Emirates; 9 Laboratory of Proteomics, Department of Human Genetics, National Institute of Health, Doutor Ricardo Jorge (INSA), 1649-016 Lisbon, Portugal; 10 Centre for Toxicogenomics and Human Health (ToxOmics), NOVA/School/Faculdade de Lisboa, 1169-056 Lisbon, Portugal

**Keywords:** prostate cancer, E3 ligase, DCUN1D1, neddylation, Wnt/β-catenin pathway

## Abstract

Defective in cullin neddylation 1 domain containing 1 (DCUN1D1) is an E3 ligase for the neddylation, a post-translational process similar to and occurring in parallel to ubiquitin proteasome pathway. Although established as an oncogene in a variety of squamous cell carcinomas, the precise role of DCUN1D1 in prostate cancer (PCa) has not been previously explored thoroughly. Here, we investigated the role of DCUN1D1 in PCa and demonstrated that DCUN1D1 is upregulated in cell lines as well as human tissue samples. Inhibition of DCUN1D1 significantly reduced PCa cell proliferation and migration and remarkably inhibited xenograft formation in mice. Applying both genomics and proteomics approaches, we provide novel information about the DCUN1D1 mechanism of action. We identified CUL3, CUL4B, RBX1, CAND1 and RPS19 proteins as DCUN1D1 binding partners. Our analysis also revealed the dysregulation of genes associated with cellular growth and proliferation, developmental, cell death and cancer pathways and the WNT/β-catenin pathway as potential mechanisms. Inhibition of DCUN1D1 leads to the inactivation of β-catenin through its phosphorylation and degradation which inhibits the downstream action of β-catenin, reducing its interaction with Lef1 in the Lef1/TCF complex that regulates Wnt target gene expression. Together our data point to an essential role of the DCUN1D1 protein in PCa which can be explored for potential targeted therapy.

## 1. Introduction

Prostate cancer is the second-most-frequent and the fifth-leading cause of cancer-related deaths in men in higher-income countries [1,2]. In lower-income countries, it is the most frequent and deadly solid cancer in men and represents a concern for the already overloaded healthcare system. In general, therapeutic strategies such as androgen ablation therapy, surgery and radiation therapy are effective for the treatment of local disease; however, very few treatment options are effective for patients with hormone-refractory metastatic disease. Deciphering pathophysiological pathways for disease development is essential to improve understanding of the mechanisms that drive PCa progression and may lead to new insights into disease development and the development of novel therapeutic approaches.

The defective in cullin neddylation 1 domain containing 1 (DCUN1D1) protein is an E3 ligase of the neddylation pathway that targets members of the cullin family of proteins [3,4,5]. It interacts with cullin proteins and facilitates the transfer of NEDD8 to them, which leads to the recruitment of ubiquitin-conjugating enzymes and the subsequent degradation of target proteins. Dysregulation of DCUN1D1, the neddylation pathway and cullin RING ligases’ (CRLs) activity can affect the degradation of specific proteins involved in cellular processes, contributing to oncogenesis, tumour growth and resistance to therapies. DCUN1D1 plays a role in tumourigenesis and progression and has been associated with poor prognosis in gliomas, lung, cervical cancer, laryngeal squamous cell carcinoma, colorectal, head and neck and prostate cancers [5,6,7,8,9,10,11,12]. Although it has been described as an oncogene in different types of cancers, the precise function of DCUN1D1 and its mechanism of action are not clearly understood. Since DCUN1D1 impacts several aspects of tumour progression, understanding its mechanism of action is key and may define it as a novel therapeutic target. In this work, we have explored the involvement and relevance of DCUN1D1 in PCa. We demonstrated that this protein is upregulated in PCa cell lines and clinical samples. Furthermore, we demonstrated that DCUN1D1 regulates PCa cell proliferation, migration and tumour formation in vivo. Using different proteomics approaches, we identified broader substrates of DCUN1D1 and identified the mechanism by which it mediates its activities in PCa, which may provide insights into its activity in general. We observed that functions associated with development were deregulated upon DCUN1D1 knockdown in PCa. Furthermore, we identified CUL3, CUL4B, RBX1, CAND1 and RPS19 as potential DCUN1D1 interactors. Our further analysis suggests that DCUN1D1 mediates preferentially the neddylation of cullins 1, 3, 4A and 5, resulting in deactivation of the WNT pathway via inactivation of β-catenin as part of its mechanism of action in PCa. Together, our data provide new insights in the role of DCUN1D1 in PCa and point to a potential therapeutic target that may lead to more precise and efficient approaches in the battle against this disease.

## 2. Materials and Methods

Cell lines authentication and culture: Cells were authenticated by the cell bank using DNA profile (STR) and cytogenetic analysis. Human PCa cell lines, 22RV1 (CRL-2505), LNCaP (CRL-1740), VCaP (CRL-2876), DU145 (HTB-81), PC-3 (CRL-1435) and normal epithelial prostate cell line and PrEC (PCS-440-010) were obtained from the American Type Culture Collection (ATCC) (Rockville, MD, USA). CW19 cells were kindly provided by Dr Steven Balk (BIDMC, HMS, Boston, MA, USA) and CL1 derived from LNCaP cells were kindly provided by Dr Sun Paik, University of California, Los Angeles. PC-3, 22RV1, CW19, VCaP, DU145 and DUCaP were grown in Dulbecco’s Modified Eagle Medium (DMEM) (Gibco, Life Technologies, Carlsbad, CA USA). LNCaP and CL1 were grown in Roswell Park Memorial Institute-1640 (RPMI-1640) medium (Sigma-Aldrich, St. Louis, MO, USA), while PrEC was grown in Prostate Epithelial Cell Basal Medium (PreEBM) (Lonza, Walkersville, MD, USA). HEK 293TT cells were grown in Dulbecco’s Modified Eagle Medium (DMEM) (Sigma-Aldrich, Darmstadt, Germany). All media were supplemented with 10% fetal bovine serum (FBS) (Biochrom AG, Berlin, Germany) and 1% penicillin (5000 μg/mL)/streptomycin (500 μg/mL) (Lonza, Walkersville, MD, USA). The cells were incubated at 37 °C in a humidified atmosphere of 5% CO_2_ until confluent.

Generation of DCUN1D1 KD PCa cells: DU145 and PC-3 DCUN1D1 KD cells were generated by transducing with the MISSION Lentiviral Transduction Particles (Sigma-Aldrich, St Louis, MO, USA) encoding the shRNA against DCUN1D1 (clone ID: TRCN0000134715) following the company’s recommendation.

Plasmids and transfection assays: The plasmids pCDNAFlag-DCUN1D1 were constructed in house by the amplification of DCUN1D1 using specific oligonucleotides (sense 5’GCGAGACGGATCCATGAACAAGTTGAAA3’ and antisense 5’CTATGACCGCGGCCGCAATCTAGAGTCGA3’ and cloned into the *Bam*HI/*Not*I restriction site of the pCDNA3.1. pcDNAMyc-Ub, which allows the expression of ubiquitin, was kindly donated by Dr Marcelo Gomes (Universidade de São Paulo, Brazil). Transfection was performed using lipofectamine plus reagent (Invitrogen) as previously described [13].

Inhibition of proteasome: Cells were plated and, once attached in the culture dishes, were treated with 1 mM of the proteasome inhibitor MG132 (Sigma-Aldrich, St. Louis, MO, USA) for 4h. DMSO (Sigma-Aldrich, Milwaukee, Germany) at the concentration of 0.1% was used as a control.

Real-time PCR: Total RNA was harvested using QIAshredder (Qiagen, Valencia, CA, USA) and the RNeasy mini kit (Qiagen) for tissue samples or cells, as recommended by the manufacturer. For evaluation of DCUN1D1 expression in human tissue samples, OriGene TissueScan Prostate Cancer Tissue Array I (OriGene Technologies, Rockville, MD, USA) was analysed by RT-PCR following the manufacturer’s protocol. All samples were normalized against GAPDH. The primers used were as follows: DCUN1D1, 5’-TCTGTGATGACCTGGCACTC-3’ (sense) and 5’-GCCATCCATGAACTCCTGTT-3’ (anti-sense); GAPDH, 5’-GTCTTCACCACCATGGAGAA-3’ (sense) and 5’-ATCCACAGTCTTCTGGGTGG-3’ (anti-sense) (IDT Technology, Coralville, IA, USA).

Immunohistochemistry analysis: Sixteen deidentified human tissue samples comprising paired cancer and normal tissues were evaluated for DCUN1D1 expression using immunohistochemistry. Ethical approval for this study was obtained from the Human Research Ethics Committee of the Faculty of Health Sciences, University of Cape Town, South Africa (approval number HREC454/2012). Immunohistochemistry was performed using the mouse monoclonal anti-DCUN1D1 antibody (Santa Cruz Biotechnology, Dallas, TX, USA) as previously described [14].

Cell migration: The assay was performed using a modified transwell chamber migration assay (BD Biosciences, Bedford, MA, USA) as previously described [13].

Proliferation and apoptosis assays: Proliferation and apoptosis assays were performed as previously described using Cell Proliferation Kit I (MTT; Roche, Basel, Switzerland) and Cell Death Detection ELISA^PLUS^ (Roche) [13,15,16,17].

Animal experiments: Eight-week-old male MF-1 nude mice, obtained from the University of Cape Town, were bred at the University’s animal facility, and housed in a pathogen-free environment. Animals were randomly divided into groups of 6 mice and subcutaneously implanted with PCa cell lines (5 × 10^6^/mice). Sample size calculation for an equal variance t-test showed the sample size needed was six per group to achieve the statistical power of 0.8. Immediately before implantation, cell lines were trypsinized and re-suspended in DMEM with 10% fetal bovine serum. Cell viability was determined by trypan blue exclusion and a single-cell suspension with 90% viability was used for implantation. Tumour size (volume) was measured on a weekly basis after implantation and the experiment was concluded when the average tumour reached 1500 mm^3^. Animals were euthanized, and the tumours were carefully dissected and weighted. All procedures with animals were reviewed and approved by the Animal Research Ethics Committee of the University of Cape Town (approved protocol number 009/062).

Microarray analysis: DU145 cells were infected with two different shRNAs against DCUN1D1 or GFP shRNA lentivirus at a multiplicity of infection of 10. Twenty-four hours post-infection, the media were replaced, and cells were incubated for an additional 48 h. RNA was collected using a QIAshredder (Qiagen) and RNeasy Mini Kit (Qiagen) and converted into cRNA according to the manufacturer’s instructions (Affymetrix, Santa Clara, CA, USA). cRNAs were hybridized to the HT U133AAofAv2 array plate (Affymetrix) and analysis was performed as described [13,15,17]. All datasets have been deposited in the Gene Expression Omnibus, (https://www.ncbi.nlm.nih.gov/geo/query/acc.cgi?&acc=GSE147820 (accessed on 1 June 2023)).

Cell lysis, co-immunoprecipitation, tryptic digestion and C18 stage tip clean-up: The cell lysis and co-immunoprecipitation assays were performed in accordance with the laboratories’ mass spectrometer (MS)-compatible immunoprecipitation protocol from the Proteomics Facility, International Centre for Genetic Engineering and Biotechnology, Trieste, Italy. Briefly, HEK 293TT cells were transfected using the calcium phosphate precipitation method and plates were incubated for 48 h until cell lysis. Transfected cells were lysed and sonicated on ice for 30 s at 80 amps using a 0.5 s cycle. Cell debris and DNA aggregates were pelleted by centrifugation at 13,200 rpm for 10 min at 4 °C. The supernatant was transferred to sterile tubes and subjected to a co-immunoprecipitation assay. Co-immunoprecipitation was performed using the EZview red anti-Flag M2 affinity gel resin containing beads with anti-Flag antibodies bound to them (Sigma-Aldrich, Germany). Gel loading tips (Corning, Costar, NY, USA ) were used to remove excess buffer solution to prevent disruption of the bead-bound pulldown products and to decrease the contaminants present in the tube prior to tryptic digestion. In-solution digestion was carried out on 20 µg total protein. Briefly, proteins were denatured with 1 mM dithiothreitol (DTT) for 1 h at room temperature with gentle agitation and alkylated for 1 h in the dark with 5.5 mM iodoacetamide (IAA). The diluted sample was then digested overnight with Trypsin (1:100 ratio) and digestion quenched with Trifluoroacetic acid (TFA) (Sigma-Aldrich, St. Louis, MO, USA). Peptide preparations were then desalted using C18 stage tips and dried before resuspension in 0.1% formic acid (FA) and 2% acetonitrile (ACN) as previously described [18].

Cell seeding and SILAC incorporation: DU145 and DU145 DCUN1D1 knockdown cell lines were used to determine the proteins that are differentially expressed following DCUN1D1 knockdown in PCa. We used the SILAC Protein Quantitation Kit DMEM (DMEM-LYS-C) (Cambridge Isotope Laboratories Inc., Cambridge, MA, USA). Media for the “light” and “heavy” samples were prepared, and then 1% penicillin (5000 μg/mL streptomycin 5000 μg/mL) (Walkersville, MD, USA) was added prior to filter sterilization of the media using 0.22 μm filters (Techno Plastic Products, Trasadingen, Switzerland). The cells were seeded onto 10 cm tissue culture dishes (Wuxi Nest Biotechnology, China) and maintained at 37 °C under humidified conditions, containing 5% CO2 for labelling incorporation.

LC-MS/MS: The MS analysis was performed at the Proteomics Facility, at the International Centre for Genetic Engineering and Biotechnology, Trieste, Italy, using the QExactive Hybrid Quadrupole-Orbitrap mass spectrometer (Thermo Fisher, Waltham, MA, USA) coupled in line with Dionex Ultimate 3500 RSnano LC systems (Dionex, Sunnyvale, CA, USA) for proteomics analysis. Liquid chromatography separation was done with an in-house-packed precolumn (100 μM ID × 20 mm) connected to a 75 μM × 500 mm analytical column packed with C18 Luna beads (5 μm diameter, 100 Å pore size; Phenomenex 04A-5452). The columns were connected to an Ultimate 3500 RS nano UPLC system (Dionex, Sunnyvale, CA, USA). A total of 1 μg of desalted peptides was loaded onto the column with the starting mobile phase of 2% ACN, 0.1% FA. Peptides were eluted with the following gradients: 10 min at 2% ACN, increased to 25% ACN for 115 min, to 35% ACN over 5 min, to 80% ACN over 5 min, followed by a column wash of 85% for 20 min. The flow rate was constant at 300 μL/min. Typical back pressure values during separation were <350 bar.

Data-dependent acquisition was performed using XCalibur in full scan mode with automatic switching between MS and MS/MS at a scan range of 300–1750 m/z and an isolation window of 2 m/z. The top 10 most abundant parent ions were selected during initial scans. The mass resolution for MS1 was 70,000, with an AGC target value of 3 × 10^6^ ions, at a maximum injection time of 250 milliseconds. The MS2 mass resolution was 17,500, with an AGC target value of 1 × 10^6^ ions, at a maximum injection time of 80 milliseconds. Peptide fragmentation was performed using high-energy collision dissociation with the normalized collision energy set at 28 and dynamic exclusion of 30 s. We used PAST version 3 (PAST3) to perform statistical analyses following immunoprecipitation-coupled MS, including univariate and multivariate tests such as the Shapiro–Wilk test for normality, the Welch F test for unequal variance, the Kruskal–Wallis test for equal medians and the principal component analysis (PCA). These were performed according to the default settings, with a significance level of 0.05, and missing values were either supported (using the mean value for the dataset) or deleted depending on the test performed and within the assumptions necessary.

Data processing and analysis: The .yep data files generated were processed using the X Tandem software version Alanine (2017.2.1.4), with the Global Proteome Machine (GPM) search engine and then run against the Ensembl human database. Default settings were used in the study, with trypsin selected for enzyme specification and carbamidomethylation of cysteine selected as a fixed modification. The variable modifications selected included single/double oxidation of methionine and tryptophan as well as deamidation of asparagine and glutamine. The dynamic range was set at 100 Daltons, with a mass tolerance of fragment ions set at 0.3 Daltons. The minimum cut-off for peptide mass was set at 150 Daltons, for parent ions it was 500 Daltons, 50 scans and 1 was set as the maximum permissible number of mixed cleavage sites. The resulting MS/MS spectra were then searched using X Tandem allowing for a 5% false positive rate (FPR).

Bioinformatics analysis: A schematic representation of the analysis performed for genomics and proteomics is shown in Supplementary Figure S1.

Differentially expressed genes in microarray analysis were identified as previously described [13]. Briefly, scanned array images were analysed using dChip and a smoothing spline normalization method. This approach allows the comparison of two groups of samples and the identification of genes that showed enrichment in a specific phenotype. To determine differential gene expression, the 90% lower confidence bound (LCB) of the fold change (FC) between the groups was evaluated and considered differentially expressed if it was higher than 1.2 as previously described [19,20,21].

Pathway analysis from microarray pathways were obtained using IPA (Qiagen, Valencia, CA, USA), which calculates *p*-value for each pathway according to the fit of a user’s data to the IPA database using one-tailed Fisher’s exact test. The functions with *p*-value < 0.001 and pathways with *p*-value < 0.05 were considered significantly affected.

The KEGG pathway database [22,23,24] was used within X Tandem to determine the pathways significantly dysregulated following IP/MS analysis based on the log (I), log (*p*) and descriptions based on database and sample frequencies. Venn diagrams were constructed using Venny 2.1.0 [25].

Perseus version 1.6.5.0 was used for data filtration and statistical analysis of the Maxquant data outputs following the SILAC/MS experiment [26]. The final MS/MS analysis was performed using the log-transformed “Ratio H/L normalized” columns, and all proteins without valid values for 100% of the replicates were removed and UniProt annotations added for further enrichment of the dataset. A two-sample t-test was used to determine whether the replicate samples were significantly different with a permutation-based FDR of 5%. Functional analysis, biological processes and pathways enriched after Silac were analysed using the Panther classification system version 14.1 [27,28,29,30], and STRING version 11 was used for functional analysis of the associations observed within the networks enriched in our dataset [31,32,33].

Western blot analysis: Western blot analyses were performed as previously described [13,16,34]. The following primary antibodies were used: DCUN1D1 (Sigma HPA035911), Nedd8 (Cell Signaling Technology, Danver, MA, USA CST2745S), Ubiquitin (Cell Signaling Technology CST39365), cullin 1 (Cell Signaling Technology CST4995), cullin 2 (Novus Biologicals, Centennial, CO, USA NBP1-67535), cullin 3 (Cell Signaling Technology CST2759S), cullin 4A (Cell Signaling Technology CST2699S), cullin 4B (Bio-Rad VMA00360) cullin 5 (Novus Biologicals NBP1-22970), APPBP1 (Cell Signaling Technology CST14321), UBA3 (Novus Biologicals NBP2-48628), UBC12 (Novus Biologicals NBP1-31459), CAND1 (Cell Signaling Technology CST8759S), RBX1 (Novus Biologicals NBP2-20113), β-Catenin (Cell Signaling Technology CST4394S) phosphor-β-Catenin (Cell Signaling Technology CST2009S) and Lef1 (Santa Cruz sc-374522).

Quantification of protein: Ratios of proteins in different cells were calculated using Image J (version 1.54d) where the intensity of the band correlates to the amount of protein. The total amount of protein present in each sample was normalized to the amount of housekeeping protein. For comparison, the amount observed in control was set up as 1.

Experimental replicates: Plasmid transfections, inhibition of proteasome, real-time PCR and Western blot analysis, migration, proliferation, and apoptosis assays were independently repeated at least three times, and a representative image of an independent experiment is presented in Section 3.

Statistical analysis was performed using the Student’s *t*-test.

## 3. Results

### 3.1. DCUN1D1 Is Upregulated in Human Prostate Cancer Cell Lines and Primary Cancer Tissue Samples

**Figure 1:**
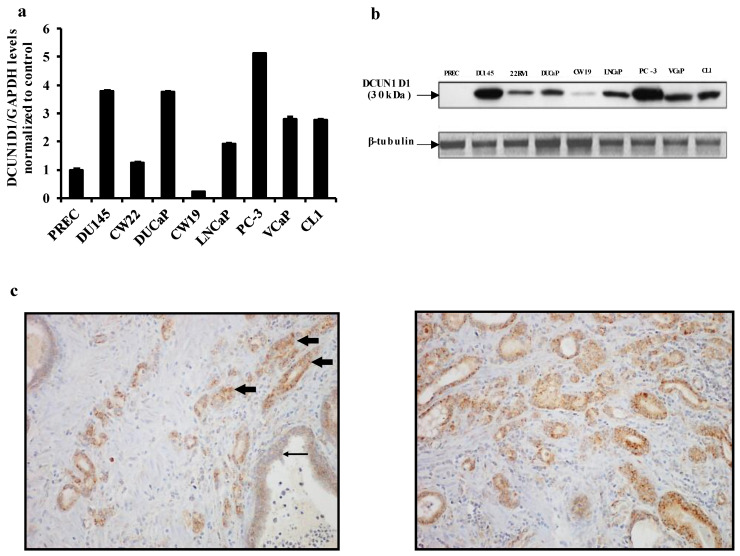
DCUN1D1 is upregulated in prostate cancer cell lines and prostate cancer tissue samples. (**a**) RT-PCR analysis of DCUN1D1 in prostate cancer cell lines. Total RNA was collected from PREC (normal), DU145, DUCaP, PC3, CL1 (androgen independent) and CW22, CW19, LNCaP and VCaP (androgen dependent cells). Normalization of each sample was carried out by measuring the amount of human GAPDH cDNA. (**b**) Western blot analysis of DCUN1D1 expression in the prostate cancer cell lines. DCUN1D1 expression was probed with anti-DCUN1D1 antibody. Normalization of each sample was carried out by β-tubulin antibodies. (**c**) Immunohistochemical staining of DCUN1D1 in representative prostate cancer cells (arrowhead) of tumour tissue exhibited intense expression compared to adjacent normal prostate tissue (long arrow). On the left, staining of DCUN1D1 in normal and prostate cancer tissue (Magnification 100×). On the right, DCUN1D1 immunohistochemical staining of prostate cancer tissue (Magnification 200×). Experiments were independently repeated three times and a representative image of an independent experiment is represented.

To understand the role of DCUN1D1 in PCa, we evaluated its expression levels in a panel of PCa cell lines that included androgen-insensitive (DU145, DUCaP, PC-3 and CL1) and androgen-sensitive (22RV1, CW19, LNCaP and VCaP) cell lines and normal prostate epithelial cells (PREC). Using RT-PCR (Figure 1a) and Western blot (Figure 1b) analysis, we observed that DCUN1D1 is upregulated in PCa cells, including androgen-insensitive PCa cells, relative to PRECs.

To further confirm these observations in primary PCa tumours, we evaluated DCUN1D1 expression levels in human tissue samples. We used the OriGene TissueScan Prostate Cancer Tissue Array I and II. Together, these two arrays contained cDNA from 69 adenocarcinoma of the prostate, 10 benign prostate hyperplasia, fifteen normal tissues, and two tissues from other disease controls (bladder carcinoma tissue). Our RT-PCR analysis demonstrated that DCUN1D1 is upregulated in 42% of the adenocarcinoma tissue samples from stages I, II and III (Table 1). Importantly, this observation was corroborated by IHC in an additional independent cohort consisting of 16 human PCa samples. We observed that seven out of the sixteen (43.7%) samples showed intense expression for DCUN1D1 that was absent from adjacent normal tissue (Figure 1c). Importantly, DCUN1D1 was also higher expressed in prostatic cancer samples with perineural invasion as compared to adjacent normal tissue (Supplementary Figure S2). In metastatic prostate cancer, elevated DCUN1D1 expression was retained (Supplementary Figure S2a,b). Together, these data indicate that DCUN1D1 is upregulated in PCa, with higher levels of expression in androgen-insensitive cells and support our hypothesis that the DCUN1D1 signalling pathway may play a role in PCa development and progression.

### 3.2. Blockage of DCUN1D1 Inhibits Proliferation and Migration and Induces Apoptosis of Prostate Cancer Cells

**Figure 2:**
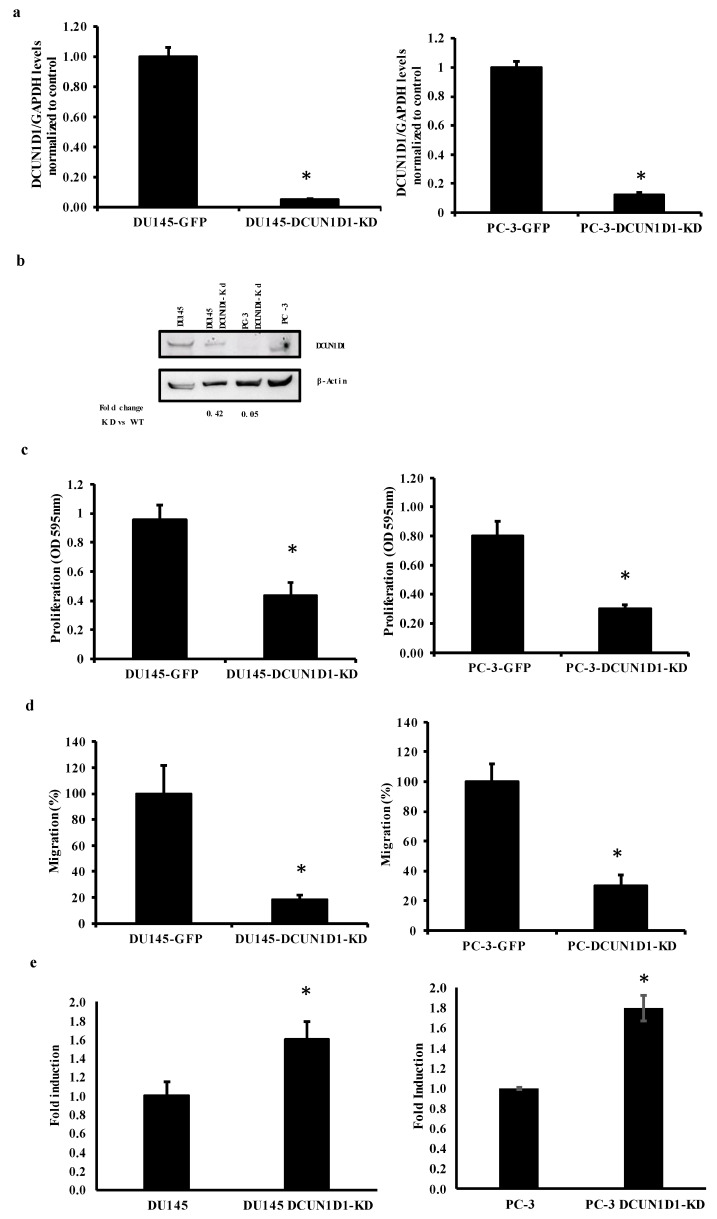
Blockage of DCUN1D1 expression inhibits proliferation and migration and induces apoptosis in prostate cancer cell lines. Prostate cancer DU145 and PC3 cell lines were infected with lentivirus-encoding shRNA against GFP and DCUN1D1. (**a**) Real-time PCR analysis of DCUN1D1 in prostate cancer cell lines 48 h post-infection. Total RNA was collected from DU145 and PC3 cell lines and normalized by measuring the amount of glyceraldehyde-3-phosphate dehydrogenase (GAPDH)-complementary DNA. (**b**) Western blot analysis of DCUN1D1 expression in DU145 and PC3 cell lines 48 h post-infection. DCUN1D1 expression was probed with anti-DCUN1D1 antibody and normalized by measuring the amount of glyceraldehyde-3-phosphate dehydrogenase (GAPDH). (**c**) Proliferations of DU145 and PC3 48 h post-infection. Data means ± s.d. of triplicate independent experiments. (**d**) Migrations of DU145 and PC3 cell lines were measured 48 h post-infection in transwell plates. Migrating cells were fixed, stained and 3–5 random microscopic fields were counted. Values shown are mean ± s.d. from a representative experiment. (**e**) Apoptosis analysis of DU145 cell lines post-infection. The Cell Death Elisa Plus kit was used to quantify apoptosis 24 h post-seeding of the cells. Data means ± s.d. of triplicate independent experiments. * *p* < 0.05, two-tailed Student’s *t*-test. Experiments were independently repeated three times and a representative image of an independent experiment is represented.

DCUN1D1 overexpression has been implicated in the proliferation and migration of cancer cells [8,35]. To understand the relevance of DCUN1D1 in PCa, we used an shRNA approach to reduce DCUN1D1 expression. DU145 and PC-3 cells were infected with lentivirus shRNA specific for DCUN1D1 (DU145-DCUN1D1-KD) and GFP (DU145-GFP) as a control. As observed in Figure 2, expression of DCUN1D1 was significantly reduced after lentivirus-specific infection. Our analysis indicates that DCUN1D1 mRNA levels are reduced by 95% and 90% in DU145 and PC-3, respectively (Figure 2a), while Western blot analysis demonstrated that DCUN1D1 expression is reduced by 96 and 98% in DU145 and PC-3 (Figure 2b). Blockage of DCUN1D1 expression strongly reduced PCa cell proliferation and migration while inducing apoptosis in PCa cells for both cell lines (Figure 2c–e). Inhibition of DCUN1D1 reduced proliferation by 54.3% and 62.5% in DU145 and PC-3, respectively (Figure 2c). Moreover, as demonstrated in Figure 2d, migration was reduced by 82% (DU145) and 75% (PC-3). Interestingly, the reduction in the expression of DCUN1D1 in PCa cells increased apoptosis levels by 61% in DU145 and 80% in PC-3 (Figure 2e). Together these data clearly indicate DCUN1D1 as a regulator of prostate carcinogenesis.

### 3.3. DCUN1D1 Is a Key Regulator of Prostate Cancer Tumour Growth In Vivo

**Figure 3:**
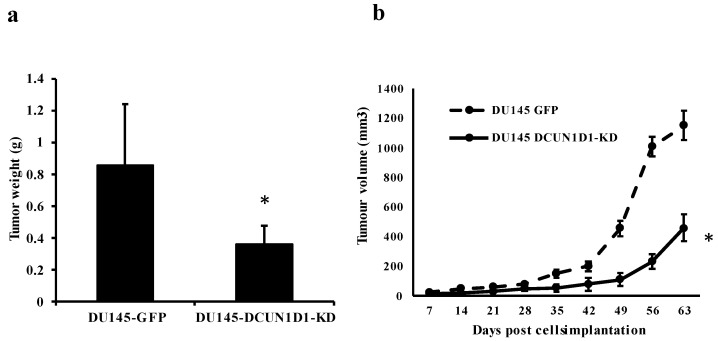
Inhibition of DCUN1D1 reduces tumour formation in MF1 nude mice. DU145 cells infected with Lv-shRNA DCUN1D1 (DU145 DCUN1D1-KD) or Lv-shRNA GFP (DU145-GFP) were implanted subcutaneously into MF1 nude mice. (**a**) The tumour weight was measured 60 days after implantation. Values are represented as mean ± s.d. of six individuals. (**b**) Tumour development throughout the study. Values are represented as mean ± s.d. of six individuals at each time point. * *p* < 0.05, two-tailed Student’s *t*-test.

After establishing DCUN1D1 as a regulator of PCa carcinogenesis, we evaluated its role in tumour formation. To determine whether inhibition of DCUN1D1 affects tumour formation in vivo, we used a xenograft mouse model MF1 where mice were subcutaneously implanted in the right flank with DU145-DCUN1D1-KD or DU145-GFP cells. Tumour development was followed, and 2 months later mice were sacrificed, examined for tumour formation and the tumours were weighted. As shown in Figure 3a, blockage of DCUN1D1 significantly reduced tumour growth with a 58% reduction in tumour weight. Likewise, tumour development was significantly delayed in DCUN1D1-KD when compared to GFP-KD control cells (Figure 3b). These data reinforce the notion that DCUN1D1 plays a key role in PCa development.

### 3.4. DCUN1D1 Interacts with CUL3, CUL4B, RBX1, CAND1 and RPS19 Proteins

After demonstrating that DCUN1D1 elicits a pivotal role in prostate cancer development, we performed a deeper analysis to explore in more detail the mechanism underlying the biological activity of DCUN1D1. To understand the role of DCUN1D1, a known E3 ligase of neddylation, in the pathways or processes linked to neddylation, we performed IP/MS analysis. IP/MS enables the identification of DCUN1D1 interactors to determine its binding partners, which will unravel the mechanisms by which it mediates its activity. HEK293T cells were transfected in duplicates with a negative control (peGFP-N3) or bait (pcDNA-Flag-DCUN1D1) plasmid, co-immunoprecipitation for either the control (Control 1 and 2) or experimental (DCUN1D1-IP1 and 2) was performed, and the pulled-down products were analysed by MS.

As shown in Supplementary Figure S3a, the percentage of unique proteins identified in each sample was as follows: Control 1 (15.2%), DCUN1D1-IP1 (28.7%), Control 2 (21.3%) and DCUN1D1-IP2 (21.6%). We identified 18 proteins that overlapped between DCUN1D1-IP1 and DCUN1D1-IP2 samples, but were absent from controls, including DCUN1D1, some known targets of DCUN1D1 such as cullin 3 (CUL3) and cullin 4B (CUL4B) and other proteins such as RBX1 and RPS19 (Supplementary Table S1).

We used the Crapome database [36] to test our dataset against a wider collection of background proteins from the aggregated negative control samples, then queried the data from each experimental sample and searched against the *H. sapiens* database. We filtered the data, based on the proteins that appeared both in our negative controls and the Crapome outputs for DCUN1D1-IP1 and DCUN1D1-IP2 samples. We performed Venn diagram analysis of the current list of proteins and found six proteins in common among DCUN1D1-IP1 and DCUN1D1-IP2 samples (Supplementary Figure S3b) which includes DCUN1D1. The five proteins identified as DCUN1D1 interactors were CUL3, CUL4B, RBX1, CAND1 and RPS19. Next, to validate our findings, we performed IP in DU145 cell extract using specific DCUN1D1 antibodies and an unspecific IgG as the control. Immunoprecipitated proteins were immunoblot against CUL3 and CUL4B (proteins identified in our IP/MS assay) and CUL1 (a known DCUN1D1 interactor). As observed in Supplementary Figure S3c, in the assay in DU145 cells, DCUN1D1 pulled-downs proteins positively reacts against CUL1, CUL3 and CUL4B. Interestingly, CUL3 and CUL4B are part of the cullin family of proteins and are known targets of neddylation by DCUN1D1 [3,4,5,37,38,39]. RBX1 is a core component of the cullin RING ubiquitin E3 ligases that is normally bound to cullins [3,37,40,41,42,43,44]. CAND1 (cullin associated and neddylation dissociated 1), is a regulator of the neddylation of cullin/RBX complexes and a regulator of the assembly of the components of the CRLs [37,42,43,45,46,47,48,49,50]. RPS19 (ribosomal protein S19) is a ribosomal protein and, although several ribosomal proteins have been identified as substrates of neddylation, this is the first time that RPS19 is described as a DCUN1D1 interactor and will be further evaluated in future research.

### 3.5. Identification of Differentially Expressed Proteins upon DCUN1D1 Inhibition in Prostate Cancer Cells

**Figure 4:**
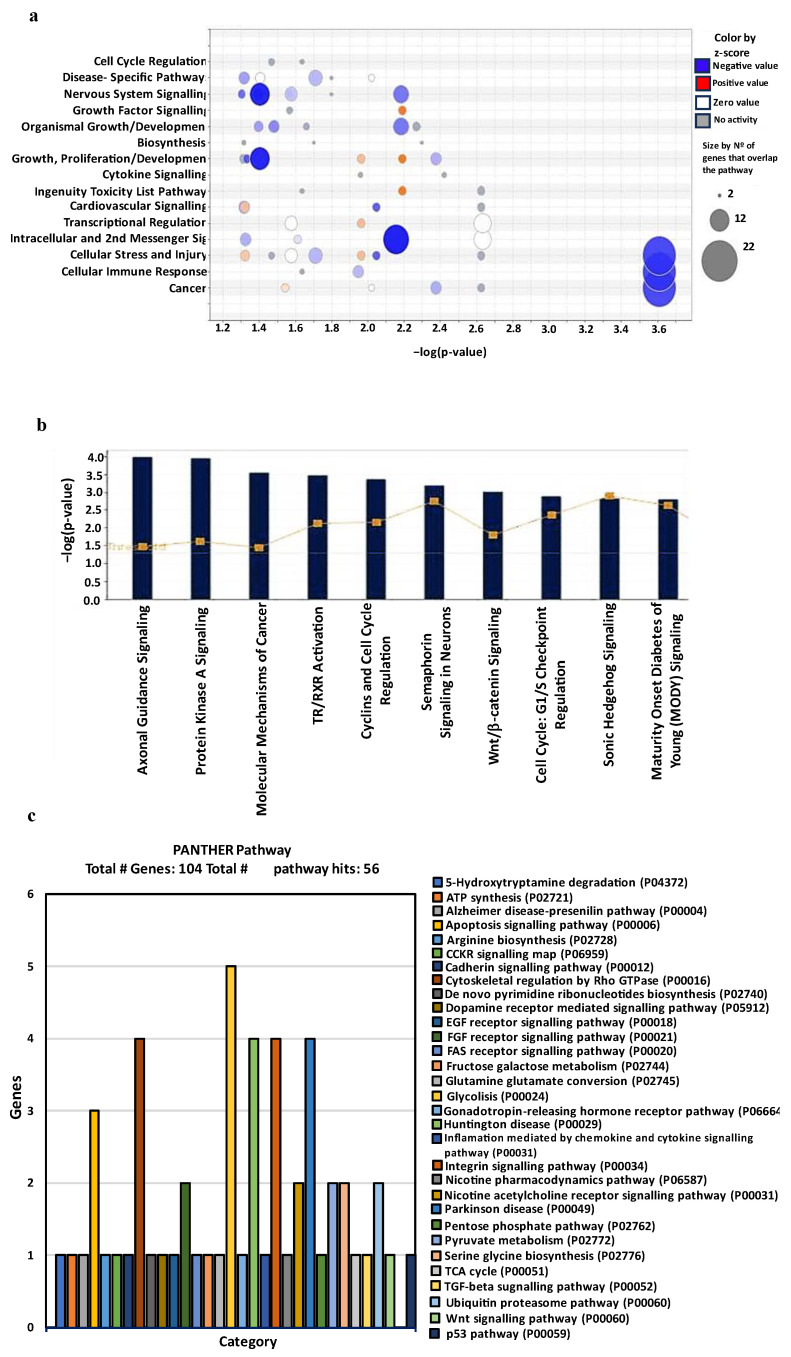
Bioinformatics analysis of functions, pathways and proteins dysregulated following DCUN1D1 knockdown in PCa cells (**a**) Enriched functions dysregulated in DU145 cells expressing the DCUN1D1 shRNA. (**b**) Top 10 pathways dysregulated following DCUN1D1 knockdown in DU145 prostate cancer cells. The list of genes up- and downregulated in DU145 cells expressing the DCUN1D1 shRNA was imported into the Ingenuity Pathway Analysis tool and canonical pathways were determined. Output represents dysregulated functions or pathways expressed as −log (*p*-value). (**c**) Bar chart of Panther pathway output from DU145 cells expressing DCUN1D1 shRNA.

Apart from identifying the direct binding of DCUN1D1 to cullins 1–5, little is known about its activity [3]. To generate new insights into the biological pathways regulated by DCUN1D1 in PCa and which genes are affected by DCUN1D1 expression, we employed a multipronged approach consisting of genomics and proteomics analysis. First, transcriptome analysis of RNA from DU145-DCUN1D1-KD was performed using Affymetrix HT U133AaofAv2 GeneChips, which contains more than 30 000 probe sets. DCUN1D1 knockdown resulted in the downregulation of 244 genes and the upregulation of 78 genes. The Supplementary Tables S2 and S3 show the lists of the top 10 downregulated and upregulated genes upon DCUN1D1 inhibition. Pathway analysis of the differentially expressed genes suggested that DCUN1D1 inhibition is associated with dysregulation of development and developmental pathways in PCa cells. Remarkably, our analysis indicated cancer, cellular immune response, cell growth, proliferation and development as the most enriched dysregulated functions upon DCUN1D1 inhibition (Figure 4a). Biological function analysis revealed most significant enrichment in functions associated with various types of solid cancer, indicating that DCUN1D1 may be involved in the development or progression of solid tumours (Supplementary Figure S4). These data corroborate the notion that this protein is an important player for cancer development. Furthermore, our pathway analysis indicated significant deregulation of axonal guidance, TR/RXR activation, semaphorin, Wnt/β-catenin and Sonic hedgehog signalling pathways (Figure 4b), suggesting that DCUN1D1 may play a role in mediating developmental functions. Interestingly, we identified deregulation of UBE2C and UBE2J2 following DCUN1D1 knockdown. These genes are both E2 ubiquitin-conjugating enzymes, which also suggests alteration of the ubiquitination pathway [51,52]. We performed ingenuity upstream regulator analysis to identify potential upstream regulators upon DCUN1D1 knockdown. We identified β-catenin as one of the more significant predicted regulators of differentially expressed genes in the DCUN1D1 knockdown of PCa cells (Supplementary Figure S5).

To further corroborate and complement our genomic data, we employed proteomic analysis using the SILAC MS/MS approach. Analysis of the duplicate samples resulted in the identification of 1443 unique peptide amino acid sequences from 3743 identified spectra. We identified 100 protein groups containing biologically relevant values, determined the relationships between these proteins identified in each replicate and performed Venn diagram analysis of our filtered samples within Perseus and observed that all proteins identified were represented in both samples (Supplementary Table S4). Additionally, for functional analysis of the samples, we analysed the properties of the samples based on these proteins followed by functional classification of our data and obtained outputs related to cellular components, molecular functions, biological processes and pathways (Supplementary Figure S5). Gene ontology (GO) analysis, empowered by the Panther classification system version 14.1, was used for functional analysis and determination of the cellular components, molecular functions, biological processes and pathways enriched in our dataset [27,28,29]. We identified molecular functions related to binding, catalytic activity and structural molecule activity. Additionally, processes related to biological regulation, cellular component organization/biogenesis, cellular processes, localization, metabolic processes and response to stimulus were also enriched (Supplementary Figure S6). Furthermore, we observed pathway classifications related to ATP synthesis, apoptosis signalling, metabolic and inflammatory pathways, the ubiquitin proteasome pathway as well as the WNT/β-catenin signalling pathway (Figure 4c). Remarkably, in both our genomics and proteomics data, the WNT/β-catenin signalling pathway was altered, indicating this pathway as a potentially relevant player in DCUN1D1-mediated phenotypic dysregulation of the molecular mechanisms of PCa.

### 3.6. DCUN1D1 Knockdown Decreases Global Neddylation, Ubiquitination and Expression of Neddylation Components Demonstrating That DCUN1D1 Shows Preferential Neddylation Activity of Cullin Proteins in PCa

**Figure 5:**
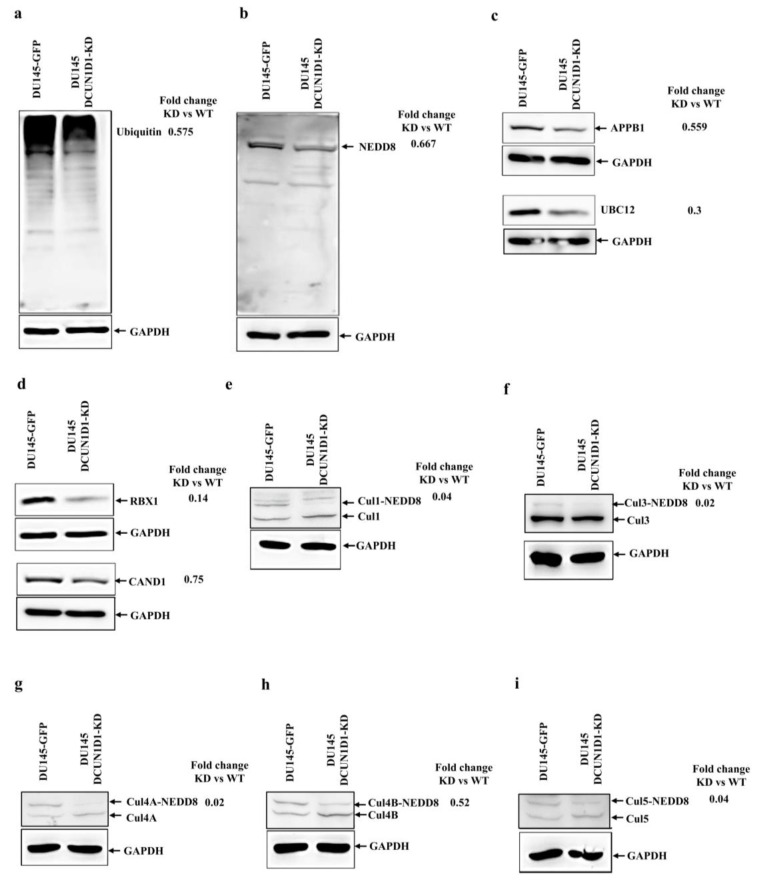
Blockage of DCUN1D1 decreases global neddylation, ubiquitination and expression of neddylation components and shows preferential neddylation activity of cullin proteins in PCa. Western blot analysis of DU145 and DU145 DCUN1D1-KD cells. Protein extracts were obtained from the cells and subjected to Western blot analysis using (**a**) anti-ubiquitin, (**b**) anti-Nedd8. Blockage of DCUN1D1 decreased the expression of the neddylation pathway components including (**c**) the E1 NAE heterodimer APPB1 (top panel) and the neddylation-conjugating enzyme, UBC12 (bottom panel) and (**d**) the cullin-associated proteins RBX1 (top panel) and CAND1 (bottom panel). Inhibition of DCUN1D1 showed preferential NEDD8 modification of the cullin family of proteins. Immunoblot analysis of DU145 and DU145 DCUN1D1 knockdown cell protein extracts using (**e**) anti-cullin 1, (**f**) anti-cullin 3, (**g**) anti-cullin 4A, (**h**) anti-cullin 4B and (**i**) anti-cullin 5. The GAPDH loading control was probed using the anti-GAPDH antibody. Experiments were independently repeated three times and a representative image of an independent experiment is represented.

The neddylation pathway is a post-translational modification process that mediates transfer of 9kDa NEDD8 to target proteins [53,54]. It also plays a role in ubiquitination through NEDD8 modification of the cullin proteins, which are scaffolding molecules of the CRLs [37,55]. DCUN1D1 is essential for neddylation, in part due to its role in the nuclear translocation of cullin 1 [3,39]. As observed in Figure 5, DCUN1D1 inhibition considerably decreases ubiquitination by 42.5% (Figure 5a) and neddylation activity by 33.3% (Figure 5b) in PCa cells. To determine whether DCUN1D1 knockdown and the reduction in neddylation was due to dysregulation in the expression of the other components of the neddylation pathway, we performed Western blot analysis. Knockdown of DCUN1D1 in DU145 PCa cells led to decreased expression (44.1%) of the E1 NAE APPBP1/UBA3 (Figure 5c, upper panel) and the neddylation conjugation enzyme UBC12 by 70% (Figure 5c, upper panel). These data demonstrate that disruption of the neddylation pathway mediated by DCUN1D1 dysregulation is key to the mechanism of action of this protein in PCa. Furthermore, we evaluated the expression levels of proteins involved in the neddylation of cullin. As demonstrated in Figure 5d, knockdown of DCUN1D1 in PCa cells decreases the expression of RBX1 and CAND1 by 85.6 and 25%, respectively. These data demonstrated dysregulation of cullin-neddylation-associated proteins following knockdown of DCUN1D1 in PCa cells. They also provided further evidence for the association of DCUN1D1 with RBX1 and CAND1, as observed previously in this study.

We evaluated whether the dysregulation in the expression of modulators of cullin neddylation impacts the expression of cullin proteins after blockage of DCUN1D1 expression. Previous studies demonstrated that RBX1and DCUN1D1 interact with cullin 1, cullin 2, cullin 3, cullin 4A, cullin 4B and cullin 5 [3,37,41]. To validate the function of these proteins as DCUN1D1 substrates and to evaluate the neddylation status of the potential DCUN1D1 cullin substrates in PCa, we performed Western blot analysis targeting cullins 1–5. We did not observe any significant changes in the expression levels of these cullins in DCUN1D1 knockdown cells; however, we observed selective decrease in NEDD8 modification in cullins 1, 3, 4A, 4B and 5 (Figure 5e–i) but not in cullin 2 (Supplementary Figure S7), reinforcing the notion that they are DCUN1D1 substrates. After DCUN1D1 knockdown, we observed decreased NEDD8 modification in cullin 1 (96%, Figure 5e), cullin 3 (98%, Figure 5f), cullin 4A (by 98%, Figure 5g), cullin 4B (48%, Figure 5h) and cullin 5 (96%, Figure 5i) when compared to the control. These data validate cullin 1, cullin 3, cullin 4A, cullin 4B and cullin 5 as DCUN1D1 substrates in PCa cells and suggests preferential cullin neddylation by DCUN1D1 as its mechanism of action in PCa.

### 3.7. The WNT Pathway Is Inhibited following Knockdown of DCUN1D1 in PCa

**Figure 6:**
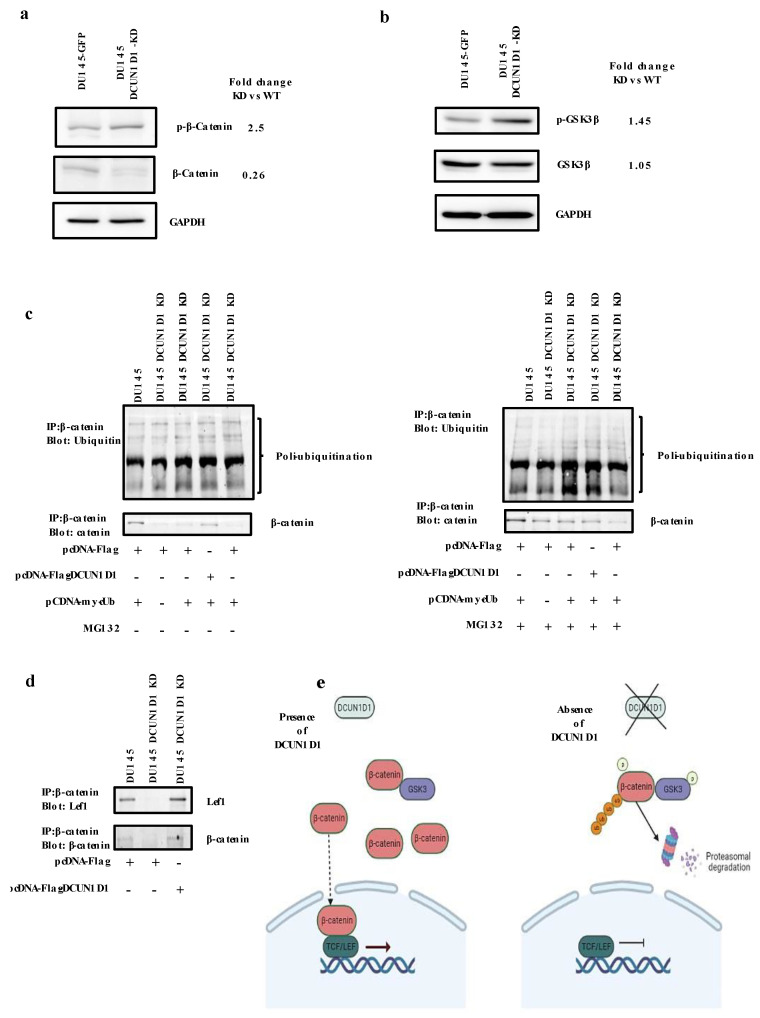
Inhibition of DCUN1D1 deactivated the WNT signalling pathway. Protein extracts containing DU145-GFP and DU145 DCUN1D1-KD cell lines were analysed using Western blot analysis. Inhibition of DCUN1D1 (**a**) increased phosphorylation of β-catenin and reduced expression of total β-catenin and (**b**) increased the phosphorylated levels of Gsk-3β. The GAPDH was used as loading control. (**c**) Blockage of DCUN1D1 inhibits ubiquitination of β-catenin. DU145 DCUN1D1-KD and DU145-GFP cells were transfected with pCDNA Myc–Ub and pCDNA Flag–DCUN1D1 expression vector or parental vector and treated with proteasome inhibitor (MG132) for 4 h. Proteins were immunoprecipitated using anti-β-catenin antibody. Ubiquitinated β-catenin was detected by using anti-ubiquitin antibody. Re-expression of DCUN1D1 protein restores β-catenin expression. (**d**) Blockage of DCUN1D1 expression inhibited β-catenin/ Lef1 interaction. Proteins from DU145 and DU145 DCUN1D1-KD transfected with pCDNA Flag–DCUN1D1 cells were immunoprecipitated using anti-β-catenin antibody. The interaction between the proteins was detected by anti- Lef1 antibodies (**e**) Schematic representation of the DCUN1D1 pathway in PCa. Experiments were independently repeated three times and a representative image of an independent experiment is represented.

Since the WNT/β-catenin pathway was predicted to be dysregulated in our microarray and proteomics analysis, β-catenin was predicted to be an upstream regulator and WNT signalling has been implicated in various cancers, we selected this pathway to further explore its involvement in the mechanism of action of DCUN1D1 in PCa. The Wnt pathway is key for development and stemness and has been associated with cancer. Interestingly, this is subject to regulation by CRL ubiquitination [56]. We evaluated the expression level of β-catenin, which is a widely characterized transcriptional co-activator of the canonical WNT pathway [57]. We observed increased phosphorylation of β-catenin (150%) and reduction in the expression level of total β-catenin (75%) in the DU145 DCUN1D1 knockdown cells when compared to the control DU145 cells (Figure 6a). Since GSK-3β plays a role in β-catenin activation, we also evaluated the activation levels of GSK-3β in DCUN1D1 knockdown cells. As observed in Figure 6b, the level of inactivated GSK-3β (i.e., in its phosphorylated form) is higher when compared to DU145 cells. The inactivation of GSK-3β leads to the inactivation of β-catenin via its degradation by the proteasome in a process mediated by its ubiquitination. In this regard, we analysed the impact of DCUN1D1 knockdown on β-catenin ubiquitination and degradation. DU145 and DU145 DCUN1D1-KD cells were transfected with a pCDNAmyc-Ub (a construct that allows the expression of ubiquitin), a DCUN1D1 expression vector (pCDNA-Flag-DCUN1D1) and a combination of both in the presence or absence of the proteasome inhibitor MG132. Western blot analysis of the immunoprecipitated β-catenin protein reveals that the ubiquitination of this protein is increased in DU145 DCUN1D1-KD cells when compared with the parental counterpart DU145 (Figure 6c). Interestingly, re-expression of DCUN1D1 in DU145 DCUN1D1-KD cells decreases the ubiquitination of β-catenin, leading to its increased expression when compared to cells deficient in DCUN1D1 (Figure 6c). These data clearly indicate that DCUN1D1 mediates the ubiquitination and degradation of β-catenin. In the activated WNT/β-catenin pathway, β-catenin is known to interact with the transcription factor Lef1 to regulate Wnt target gene expression. We investigated whether expression of DCUN1D1 interferes with β-catenin/ Lef1 interaction. Immunoprecipitation of β-catenin followed by detection with anti- Lef1 antibodies was performed. As observed in (Figure 6d), β-catenin and Lef1 are not co-immunoprecipitated in DU145 DCUN1D1 KD cells. Strikingly, when DCUN1D1 expression is restored, the interaction of β-catenin/ Lef1 is recovered (Figure 6d). These data indicate deactivation of the WNT signalling pathway following knockdown of DCUN1D1. In the absence of stimulation of the WNT pathway by ligand binding, cytoplasmic β-catenin is phosphorylated and then targeted for proteasomal degradation, repressing the expression of WNT target genes [56,57]. Our data clearly demonstrate that the blockage of DCUN1D1 led to the deactivation of the WNT pathway, validating its role in the mechanism of action of DCUN1D1.

## 4. Discussion

DCUN1D1 is an E3 ligase of the neddylation pathway and has been reported to be upregulated and associated with aggressive phenotypes and poor clinical outcomes in several types of cancer, particularly head and neck, lung, cervical and ovarian cancer as well as gliomas [3,4,5,8,9,10,11,12]. In this study, we demonstrated that DCUN1D1 is upregulated in PCa. This result confirms protein expression data from the Human Protein Atlas database that indicates highest DCUN1D1 protein expression among cancers in PCa (https://www.proteinatlas.org/ENSG00000043093-DCUN1D1/pathology (accessed on 14 October 2022)) and corroborates previous reports [6,7]. Previous studies demonstrated that DCUN1D1 is associated with the progression and prognosis of PCa and plays a tumour promotive role [6,7]. We now performed a more in-depth analysis and generated novel insights to better understand the molecular mechanism of DCUN1D1 in PCa. Our analysis indicates that this protein plays a key role in hallmarks of cancer such as induction of proliferation, migration and apoptosis, as well as in inducing tumour formation in xenograft mice models, which is in line with the role of this protein in different other types of cancer [8,35,58,59]. Some of these functions have been observed in prior PCa studies as well [6,7]. We have demonstrated that in PCa, blockage of DCUN1D1 inhibits tumour growth in a nude mice model and that, upon inhibition of DCUN1D1, PCa cells lose their ability to proliferate and migrate and they undergo apoptosis. DCUN1D1 clearly functions as an oncogene in PCa and dysregulation in its expression is detrimental to cancer. As an E3 ligase of the neddylation pathway, DCUN1D1 may be an optimal target for molecular target-based therapeutics [60,61]. In fact, proteasome and neddylation pathway inhibition are among emerging cancer treatment strategies with bortezomib and the NAE inhibitor MLN4924, showing promising results [62,63,64,65]. Furthermore, the development of an inhibitor that blocks the interaction of DCUN1D1 with the neddylation-conjugating enzyme, UBC12, resulting in selective inhibition of cullin 3 neddylation, is ongoing [66]. In this context, understanding the mechanism of action of DCUN1D1 is extremely relevant. In this work, we employed genomics, high throughput proteomics and molecular biology analysis to understand the mechanism of action of DCUN1D1 in PCa. We used microarrays, IP/MS and label-based relative quantitative proteomics to identify proteins involved in the mechanism of action of DCUN1D1.

IP/MS indicated DCUN1D1 interaction with cullin proteins and cullin-associated neddylation components which impacts the ubiquitin proteasome pathway. Our analysis identified 3 members of the ubiquitin CRLs: BCR E3 ligases (BTB-CUL3-RBX), DCX E3 ligases (DDB1-CUL4-XBOX) and the ECS E3 ligases (ELONGIN BC-CUL2/5-SOCS box) and RPS19 (ribosomal protein S19) as DCUN1D1 interactors. Furthermore, using SILAC quantitative proteomics analysis, we identified proteins differentially expressed upon DU145 DCUN1D1 knockdown. Our analysis demonstrated changes in the proteome classified as cellular components, organelles and structure molecule activity. Interestingly the biological processes dysregulated upon DCUN1D1 knockdown include biological regulation, cellular processes, localization, metabolic processes and response to stimulus. In addition to the implication of metabolic processes and inflammatory responses, we observed dysregulation of the WNT pathway in both the transcriptome and proteome analysis. Moreover, our pulldown assay identified cullins and cullin neddylation components as substrates for DCUN1D1. Additionally, we identified the ubiquitin-activating enzyme UBA1 as a DCUN1D1 interactor, providing further evidence for the association of UBA1 with the neddylation pathway as demonstrated previously [67]. Other than RPS19, we also observed the dysregulation of potential neddylation substrates including ribosomal proteins such as RPS6, RPS16 and RPL27, following IP/MS analysis of DCUN1D1 pulldown products which will be analysed in detail in future works for its functional implication.

Based on the data obtained, we propose a mechanism of action for DCUN1D1. Blockage of DCUN1D1 leads to dysregulation of the neddylation pathway, ubiquitination and deactivation of the WNT/β-catenin pathway via the phosphorylation of GSK and β-catenin. β-catenin phosphorylation leads to its degradation and impedes its interaction with the Lef1/TCP complex (Figure 6e). Additionally, although DCUN1D1 has been demonstrated to neddylate cullins 1–5, our data suggest selective DCUN1D1-mediated cullin neddylation in PCa [3,4]. This is the first description of DCUN1D1-mediated preferential neddylation of cullin proteins in PCa. Moreover, we observed the deactivation of the WNT/β-catenin pathway following blockage of DCUN1D1. The WNT/β-catenin pathway has been shown to play a critical role in cell proliferation, embryonic development and the pro-inflammatory phenotype in the tumour environment [68,69,70], and is consistent with our observations, implicating it in the mechanism of action of DCUN1D1.

This study has provided further insights into the cellular activities of the DCUN1D1-regulated pathway in PCa. Analysis of DCUN1D1 binding partners identified cullin proteins as the target proteins of DCUN1D1 that we postulate may be the primary targets of DCUN1D1. In addition, key cellular pathways were implicated including proteasome degradation, transcription, translation, metabolism and inflammation. We validated the role of cullin 3, cullin 4B and cullin 5 neddylation in the mechanism of DCUN1D1 in PCa, demonstrating preferential decreases in the cullin neddylation of cullins 1, 3, 4A, 4B and 5, and the deactivation of the WNT/β-catenin pathway via GSK-3 inactivation. In fact, our findings corroborated the notion that the Wnt/β-catenin pathway is regulated by CRL ubiquitination [56] and plays a role in PCa. In conclusion, DCUN1D1 mediates its mechanism of action in PCa through the dysregulation of the neddylation and ubiquitination pathways and activation of WNT/β-catenin pathway.

## 5. Conclusions

This study provides comprehensive evidence supporting the role of DCUN1D1 as an oncogene in PCa. While upregulated in PCa, DCUN1D1 is involved in crucial cancer hallmarks such as proliferation, migration, apoptosis and tumour growth. Our findings suggest that DCUN1D1 is a potential target for targeted therapeutics, particularly in combination with proteasome and neddylation pathway inhibitors. We revealed the interaction of DCUN1D1 with cullin proteins and components of the ubiquitin proteasome pathway, as well as the dysregulation of the WNT/β-catenin pathway. The proposed mechanism of action involves the dysregulation of neddylation, ubiquitination and deactivation of the WNT/β-catenin pathway, highlighting the critical role of DCUN1D1 in PCa progression, which bring insights into the cellular activities and potential therapeutic targeting of DCUN1D1 in PCa.

## Figures and Tables

**Table 1:** Real-time PCR analysis of DCUN1D1 gene expression in human prostate cancer tissue.

Tissue Type	Number of Upregulated Samples/Number of Samples (%) ^1^	Fold Induction Compared to Normal Prostate Tissue ^2^
Adenocarcinoma of prostate (stage I)	9/26 (34.6%)	2 to 10
Adenocarcinoma of prostate (stage II)	13/29 (44.8%)	5 to 50
Adenocarcinoma of prostate (stage III)	7/16 (43.75%)	20 to 2000
BPH	0/10 (0%)	0
Carcinoma of bladder, transition tissue	0/2 (0%)	0

^1^ DCUN1D1 is upregulated in 29/69 (42%) of clinical samples analysed. ^2^ The results are shown as fold of induction compared with the values obtained in normal prostate.

## Data Availability

All date sets have been deposited in the Gene Expression Omnibus, https://www.ncbi.nlm.nih.gov/geo/query/acc.cgi?&acc=GSE147820 (accessed on 1 June 2023).

## Supplementary Materials

The following supporting information can be downloaded at: https://www.mdpi.com/article/10.3390/cells12151973/s1.
