# Supplementary material for: DCUN1D1 Is an Essential Regulator of Prostate Cancer Proliferation and Tumour Growth That Acts through Neddylation of Cullin 1, 3, 4A and 5 and Deregulation of Wnt/Catenin Pathway
Source: Cells. 2023 Jul 31;12(15):1973. doi: 10.3390/cells12151973 (PMC10417424; doi:10.3390/cells12151973)
Supplement: Supplementary file 1 [file cells-12-01973-s001.zip › cells-2499010-supplementary.pdf]

Supplementary Figure 1

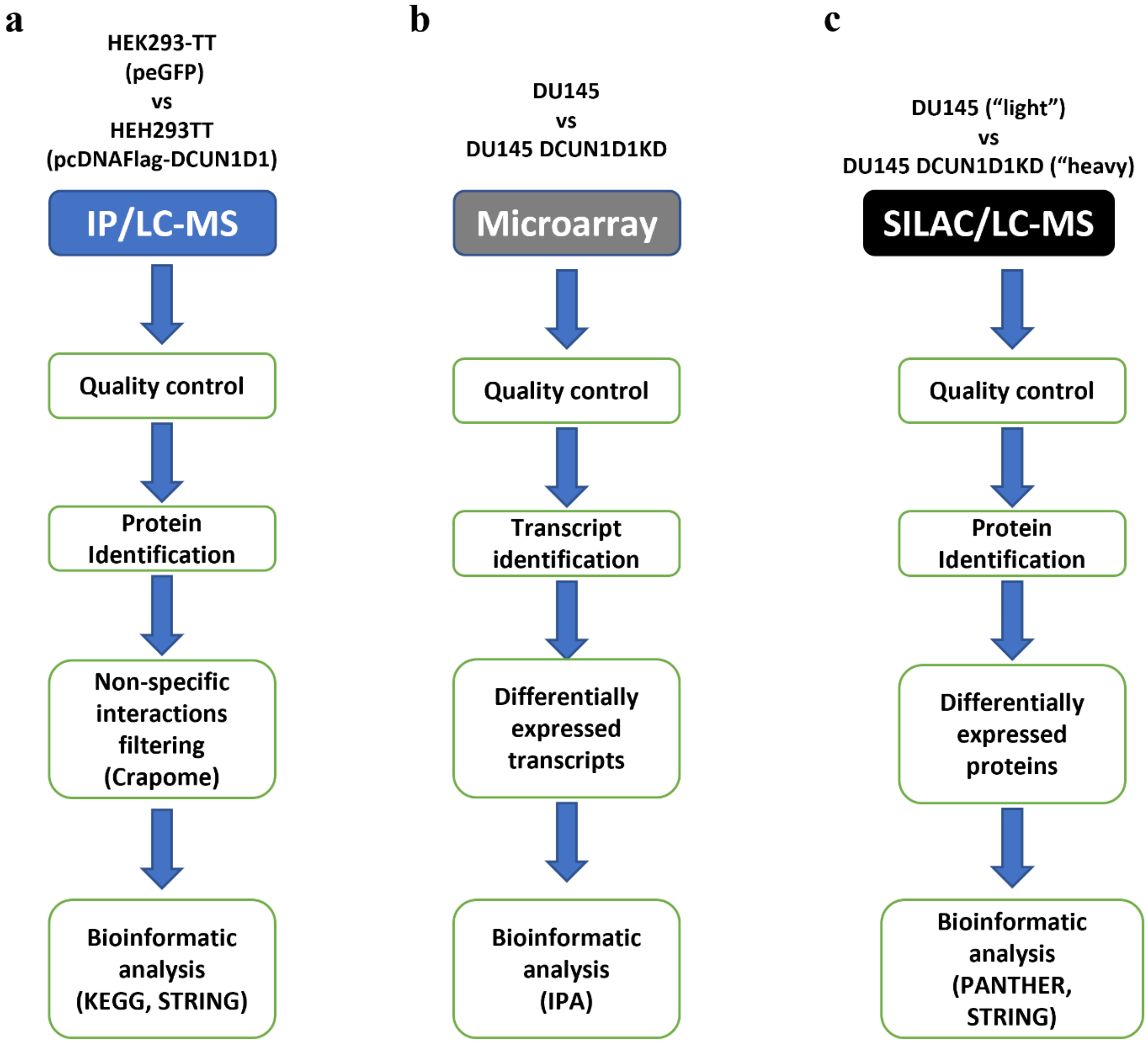

Supplementary Figure 1. Schematic representation of the analysis performed. Workflow representing the main analyses performed in: **(a)** IP/LC-MS, used for the identification of DCUN1D1 binding partners**, (b)** microarray and **(c)** SILAC/LC-MS, used for the identification of proteins and transcripts differentially expressed upon DCUN1D1 inhibition.

Supplementary Figure 2

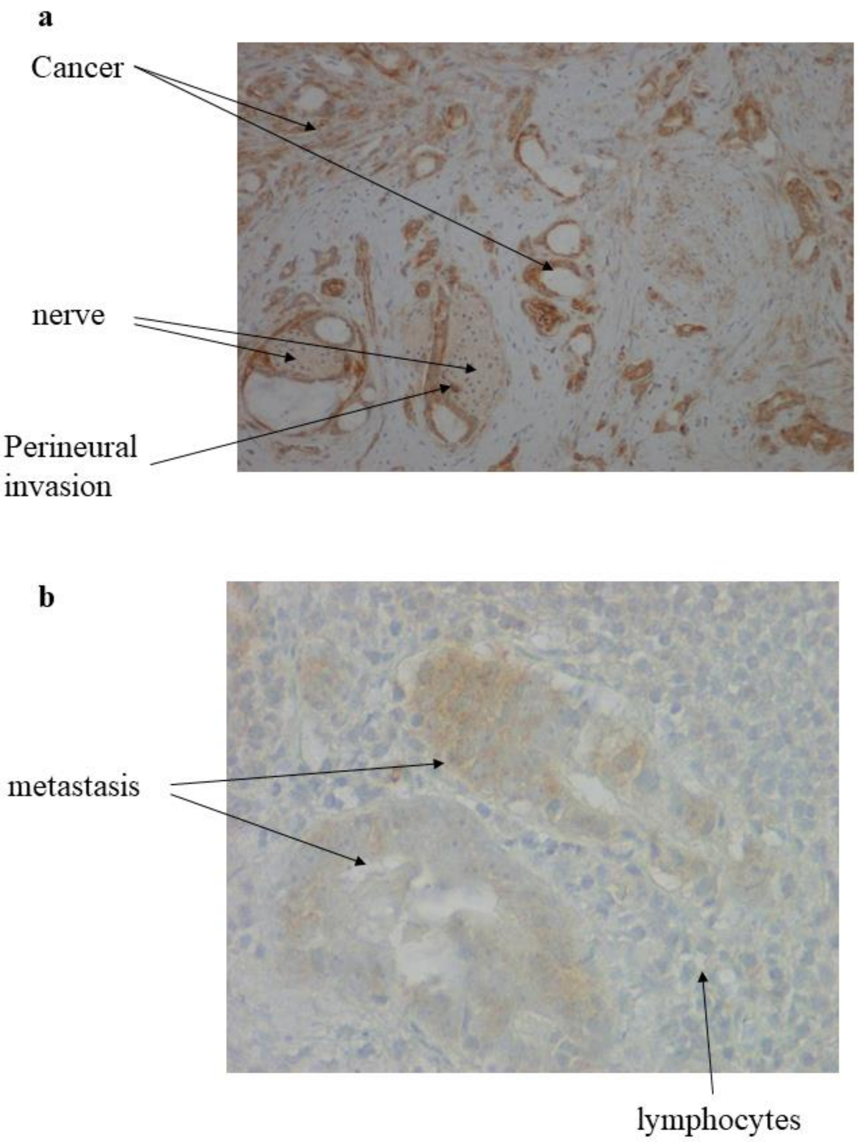

Supplementary Figure 2. DCUN1D1 is upregulated in metastatic prostate cancer tissue. Immunohistochemical staining of DCUN1D1 in representative prostate cancer biopsies. Staining of DCUN1D1 in perineural invaded (**a**) and (**b**) metastatic samples.

Supplementary Figure 3

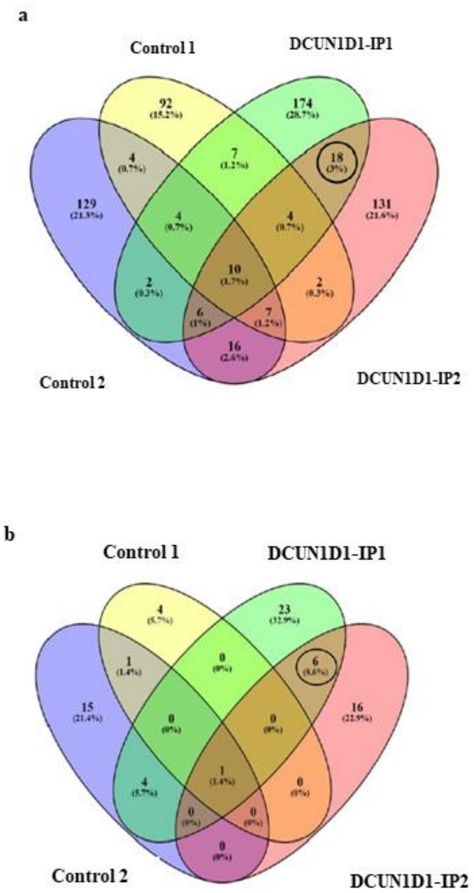

Supplementary Figure 3. Analysis of the proteins identified for each sample to determine the relationships between the samples DCUN1D1-IP1 and IP2. HEK293T cells were transfected in duplicates with either a negative control (peGFP-N3) or pcDNA-Flag-DCUN1D1 plasmid, submitted to co-immunoprecipitation for either the control (Control 1 and 2) or experimental (DCUN1D1-IP1 and 2), and analyzed by MS. Venn diagrams were generated using Venny 2.1.0 software. (**a**) Venn diagram distinguishing between proteins within a sample and those shared between a subset of the samples. (**b**) Venn diagram showing relationship between the samples

post-filtering. Data was filtered using the Crapome version 1.1, workflow 1, then we queried the data from each sample DCUN1D1-IP1 and 2 and searched against the H. sapiens database.

Supplementary Figure 4

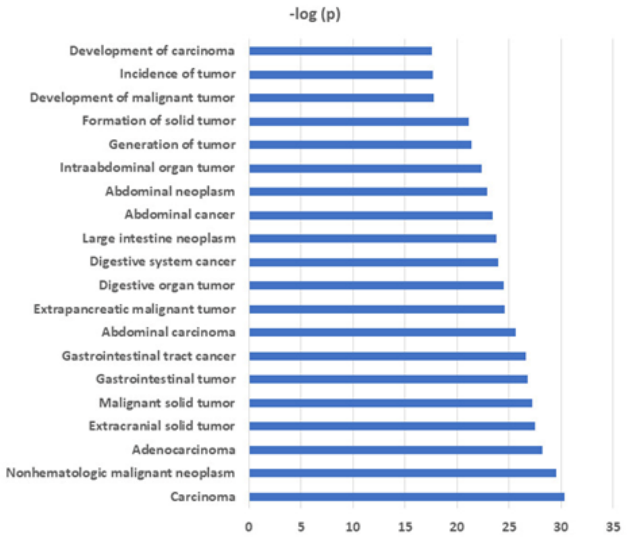

Supplementary Figure 4. Biological function analysis mediated by DCUN1D1 in PCa cells. Dysregulated cancer functions in DU145 cells expressing the DCUN1D1 shRNA. Output is expressed as –log (p-value).

Supplementary Figure 5

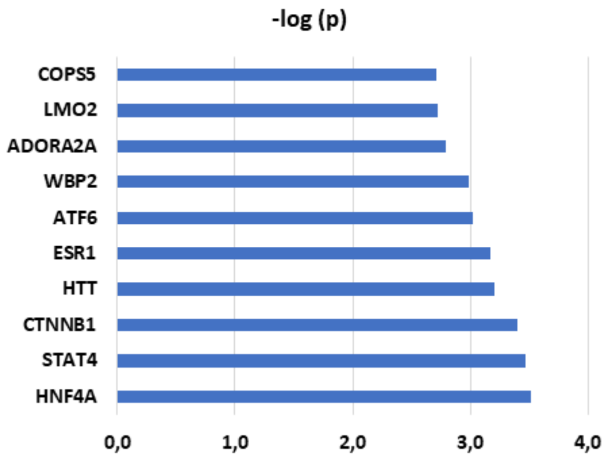

Supplementary Figure 5. Ingenuity Upstream Regulator Analysis mediated by DCUN1D1 in PCa cells. Regulators affected by expression of DCUN1D1 shRNA in DU145 cells. Output is expressed as –log (p-value).

Supplementary Figure 6

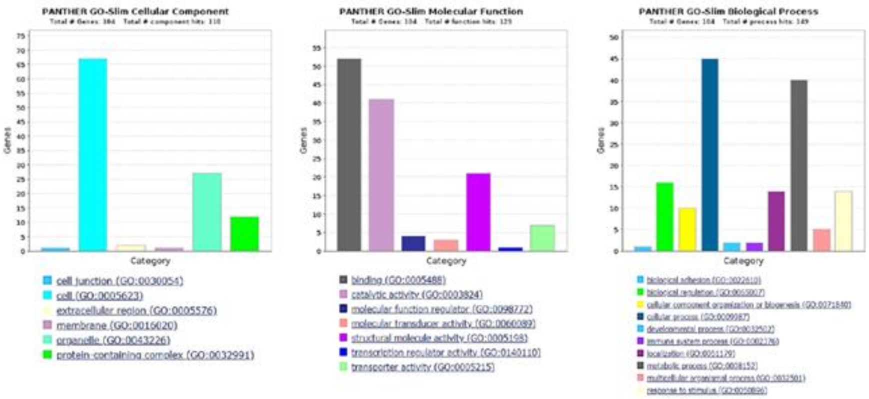

Supplementary Figure 6. Bioinformatics analysis of proteins dysregulated following DCUN1D1 knockdown PCa cells. Functional classification of cellular components, molecular functions, biological processes and pathways in DU145 cells expressing the DCUN1D1 shRNA. Bar charts

of Panther GO-Slim outputs. (Left) Experimental data classified according to cellular components, (centre) experimental data classified according to molecular functions and (right) experimental data classified according to biological processes.

Supplementary Figure 7

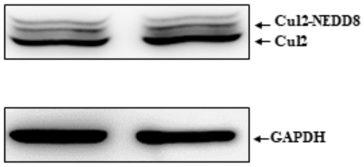

Supplementary Figure 7. Blockage of DCUN1D1 does not affect neddylation of Cul2. Immunoblot analysis of DU145 and DU145 DCUN1D1 knockdown cell protein extracts using The GAPDH loading control was probed using the anti-GAPDH antibody. All experiments were independently repeated three times and the blots correspond to a representative image of an independent experiment.

Supplementary table 1: Details on the 18 proteins found to overlap between samples DCUN1D1 IP1 and DCUN1D1 IP2

Protein Name	DCUN1D1 IP1 Accession number	DCUN1D1 IP1 Unique Peptides	DCUN1D1 IP2 Accession number	DCUN1D1 IP2 Unique Peptides
**DCUN1D1**	ENSP00000292782	14	ENSP00000292782	11
**CAND1**	ENSP00000442318	13	ENSP00000442318	25
**CUL3**	ENSP00000264414	10	ENSP00000264414	9
**RPS19**	ENSP00000221975	5	ENSP00000221975	2
**CUL4B**	ENSP00000360373	4	ENSP00000360373	3
**RBX1**	ENSP00000216225	2	ENSP00000216225	2
**BAT2L1**	ENSP00000384606	2	ENSP00000384606	1
**C1orf77**	ENSP00000357683	1	ENSP00000357683	2
**MCAT**	ENSP00000290429	1	ENSP00000290429	1
**C10orf103**	ENSP00000394678	1	ENSP00000394678	1
**COL19A1**	ENSP00000316030	1	ENSP00000316030	1
**TMEM14A**	ENSP00000211314	1	ENSP00000211314	1
**COL1A2**	ENSP00000297268	1	ENSP00000297268	2
**COL15A1**	ENSP00000364140	1	ENSP00000364140	1
**G6PD**	ENSP00000377192	1	ENSP00000377192	1
**ACE2**	ENSP00000252519	1	ENSP00000252519	1
**COL13A1**	ENSP00000381949	1	ENSP00000381949	1
**PITRM1**	ENSP00000370367	1	ENSP00000224949	1

Supplementary table 2: List of top 10 upregulated genes after inhibition of DCUN1D1

Gene	Description	Fold change
**1** ATP6AP2	ATPase, H+ transporting, lysosomal accessory protein 2	19.97
**2** EEF2	Eukaryotic translationelongation factor 2	6.3
**3** UBE2C	Ubiquitin-conjugatingenzyme 2C	5.9
**4** SPTBN1	Spectrin, beta, nonerythrocytic1	4.4
**5** BAT2D1	BAT2 domaincontaining 1	4.04
**6** TNPO1	Transportin 1	3.85
**7** SF3B1	Splicing factor 3b,subunit 1, 155kDa	3.68
**8** CADM1	Cell adhesion molecule 1	3.59
**9** SH3GLB1	SH3-domain GRB2-like endophilin B1	3.48
**10** HAX1	HCLS1 associatedprotein X-1	3.24

Supplementary table 3: List of top 10 downregulated genes after inhibition of DCUN1D1

Gene	Description	Fold change
**1** UBE2J2	Ubiquitin-conjugating enzyme E2, J2	-9.5
**2** BAT2	HLA-B associated transcript 2	-8.46
**3** ZFPM1	Zinc finger protein, multitype 1	-6.97
**4** CLDN6	Claudin 6	-6.73
**5** TNPO2	Transportin 2	-6.4
**6** NMNAT3	Nicotinamide nucleotide adenylyltransferase 3	-5.93
**7** EPHB4	EPH receptor B4	-5.38
**8** ACAA2	Acetyl-Coenzyme A acyltransferase 2	-5.36
**9** ACAD10	Acyl-Coenzyme A dehydrogenase family, member 10	-5.04
**10** FAM109A	Family with sequence similarity 109, member A	-5.01

Supplementary table 4: List of 100 proteins found across the replicate samples

Protein name			
EIF3C	HSP7C	GRP75	1433E
PHGDH	PABPC1	RS19	RPS14
ACTN4	EF2	RPS9	RS13
HNRPQ	KPYM	RS10	RL7A
LDHA	ENPL	IDHP	RS6
DHE3	DDX5	TCPG	E5RI99
ALDOA	TCPA	EFTU	PPIA
ANXA1	PGAM1	SYAC	RACK1
G3P	FLNA	GDIB	EF1A3
AT1A1	UBA1	TCPQ	TBB4B
ALDH2	ROA2	HNRPF	SLC25A3
ADT2	RS3	SYYC	HNRNPU
EIF2S1	SAHH	XPO2	SRSF2
CDK1	ATPA	TERA	EIF4G1
ATPB	MOES	EIF3B	PRDX1
ENO1	RPL13	MYL6	TIF1B
NPM	VARS	ACTB	IMB1
P4HB	CAD	IF4A1	PLEC
ANXA2	TKT	RS20	PCBP2
TUBB	PDIA3	RS3A	TBA1C
HS90A	PPP2R1A	RPL15	PDC6I
HS90B	GLYM	RL27	NAA15
ODPA	HSPA4	HNRPK	NSF1C
RPSA	MYH9	RS8	RUVBL2
BIP	PRS7	RS16	TLN1
